# Organ Donation in Croatia: The Importance of a National Champion, a Comprehensive Plan, and International Collaborations

**DOI:** 10.3389/ti.2023.11011

**Published:** 2023-05-25

**Authors:** Jasmine Mah, Charlotte Johnston-Webber, Apostolos Prionas, Mirela Bušić, Simon Streit, George Wharton, Elias Mossialos, Vassilios Papalois

**Affiliations:** ^1^ Department of Medicine, Dalhousie University, Halifax, NS, Canada; ^2^ Department of Health Policy, London School of Economics and Political Science, London, United Kingdom; ^3^ Department of Surgery, Imperial College, London, United Kingdom; ^4^ Department of General Surgery, Whipps Cross Hospital, Barts Health NHS Trust, London, United Kingdom; ^5^ Ministry of Health, Zagreb, Croatia; ^6^ Institute of Global Health Innovation, Imperial College, London, United Kingdom; ^7^ Renal and Transplant Unit, Hammersmith Hospital, Imperial College Healthcare NHS Trust, London, United Kingdom

**Keywords:** organ donation, organ transplantation, transplantation policy, transplant system, Croatia

## Abstract

The Republic of Croatia is a global leader in organ donation and transplantation despite having fewer resources and more modest healthcare expenditures than other countries in the European Union. The results of an extensive literature review were combined with expert input in an iterative multi-step data collection and evaluation process designed to assess trends in Croatian organ donation and transplantation and identify key elements, policy changes, and drivers of the system that have contributed to its success. Multiple sources of evidence were used in this study, including primary documents, national and international transplantation reports, and insights from critical informants and content experts. The results highlight several key organizational reforms that have substantially improved the performance of the Croatian transplant program. Our findings emphasize the importance of strong central governance led by an empowered national clinical leader acting under the direct auspices of the Ministry of Health and a comprehensive and progressive national plan. The Croatian transplant system is notable for its integrated approach and efficient manner of managing scarce health resources. Collectively, the results suggest that Croatia has become nearly self-sufficient due to its systematic implementation of the guiding principles for organ donation and transplantation.

## Graphical Abstract

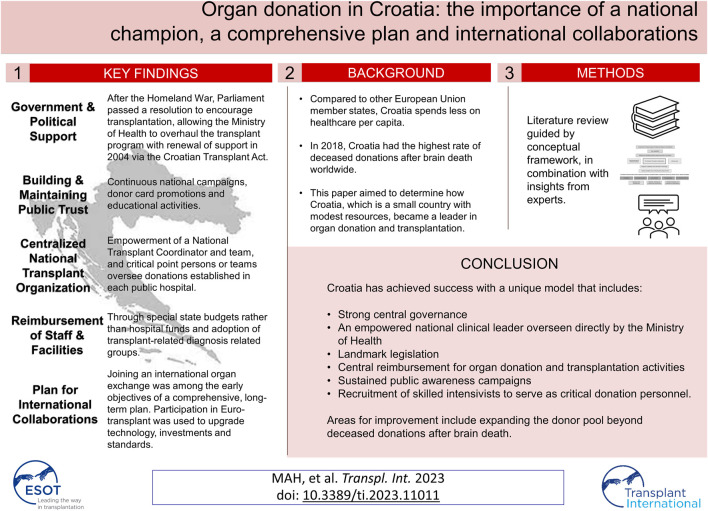

## Introduction

Despite a relatively poor economy compared to other European Union (EU) member states and a 12% reduction in its gross domestic product since the 2008 worldwide economic crisis (1), Croatia has become a global leader in organ donation and transplantation. In 2018, Croatia had the highest rate of deceased donations after brain death (DBD) worldwide, reported at 40.24 per million population (pmp). Total deceased donations in Croatia were second only to those recorded in Spain (which also supports a robust donation after circulatory death (DCD) program). Croatia ranks sixth overall for total transplant activity worldwide (2).

This study aimed to determine how Croatia, which is a small country with modest resources, became a leader in organ donation and transplantation. Our goal is to provide information that may be useful for countries with similar resource constraints that nonetheless wish to improve their transplant systems. We intended to assess trends in transplantation and organ donation from 2000 to 2018/2019 (i.e., before the onset of the COVID-19 pandemic) and identify key elements, policy changes, and drivers of the Croatian transplantation system that have contributed to its success. Table 1 provides an overview of key statistics on the Croatian healthcare system and population health status, including health spending *per capita*, public versus private health expenditure and number of people on renal replacement therapy.

**TABLE 1:** Health system financing and population health in Croatia: key statistics.

Healthcare system	References
• Mandatory health insurance with healthcare financed by the Croatian Health Insurance Fund (Hrvatski zavod za zdravstveno osiguranje or [HZZO])	(3)
• Health spending *per capita*, 1392 EUR; EU average, 3523 EUR	(3)
• Health spending as a percentage of the GDP, 7.0%; EU average, 9.9%	(3)
• Public spending as a percentage of the total healthcare expenditures, 81.9%; EU average, 79.7%	(3)
• Out-of-pocket payments as a percentage of total healthcare expenditures, 11.5%; EU average, 15.4%	(3)
• Percentage of the population reporting an unmet need for medical care, 1.4%; EU average, 1.7%	(3)
Health status	
• Percentage of the population over 65 years of age, 21.0%; EU average, 20.6%	(3)
• Life expectancy, 77.8 years; EU average, 80.6 years	(3)
• Percentage of adults that smoke daily, 22%, OECD average, 16.5%	(3, 4)
• Percentage of adults that binge drink alcohol, 16.6%	(3)
• Percentage of adults that are overweight or obese (BMI >25), 23%; OECD average, 56.4%	(3, 4)
• Fraction of patients maintained on renal replacement therapy, 622	(5)

EUR, euro; EU, European Union; OECD, Organization for Economic Co-operation and Development; BMI, body mass index.

## Materials and Methods

Three main sources of evidence were used to develop this case study. First, a targeted narrative literature review was conducted based on the guidelines provided by the conceptual framework developed by Johnston-Webber et al. (6). The literature searches focused on publications listed in the Medline and Web of Science databases. We identified peer-reviewed publications using combinations of the keywords “organ donation and transplantation” and “Croatia.” While the search was not limited by year of publication, we excluded publications published in languages other than English or Croatian. We also performed hand-searches of the references listed in the original selections based on their relevance to study objectives and areas identified by the aforementioned conceptual framework. An additional search of grey literature provided additional important information; this information was located by searching Google Scholar, government websites (the Croatian Ministry of Health), and websites of key international organ donation and transplantation organizations. Critical documents were retrieved, including guidelines and resources relevant to Eurotransplant, the South-Eastern Europe Health Network (SEEHN), the European Directorate for the Quality of Medicines & Healthcare, and the Global Observation on Donation and Transplantation (GODT).

We also collected evidence from a panel of international experts in healthcare systems policy and transplantation. The findings from the literature review were initially compiled by one researcher (JM) and were then modified *via* an iterative process following rounds of written and verbal feedback from the expert panel; this included the involvement of the National Transplant Coordinator (NTC) (also an author) providing leadership and feedback throughout. In addition to providing feedback on our findings, the experts also suggested additional resources or documents that might be included in the text.

This approach to the collection and synthesis of data was designed to provide a holistic overview of the Croatian organ donation and transplantation program; information from both quantitative and qualitative sources was included. Therefore, in addition to efforts to assess quantitative trends in key transplant indicators, the subjective experiences of people who worked in (or closely with) the Croatian transplantation system were also considered. The analysis was designed to include all factors that may have had an impact on the performance of the organ donation and transplantation program. The analysis focused on structures, processes and distinctive features of the system corresponding to domains of the framework, rather than performance in relation to health outcomes or health system goals.

**FIGURE 1:**
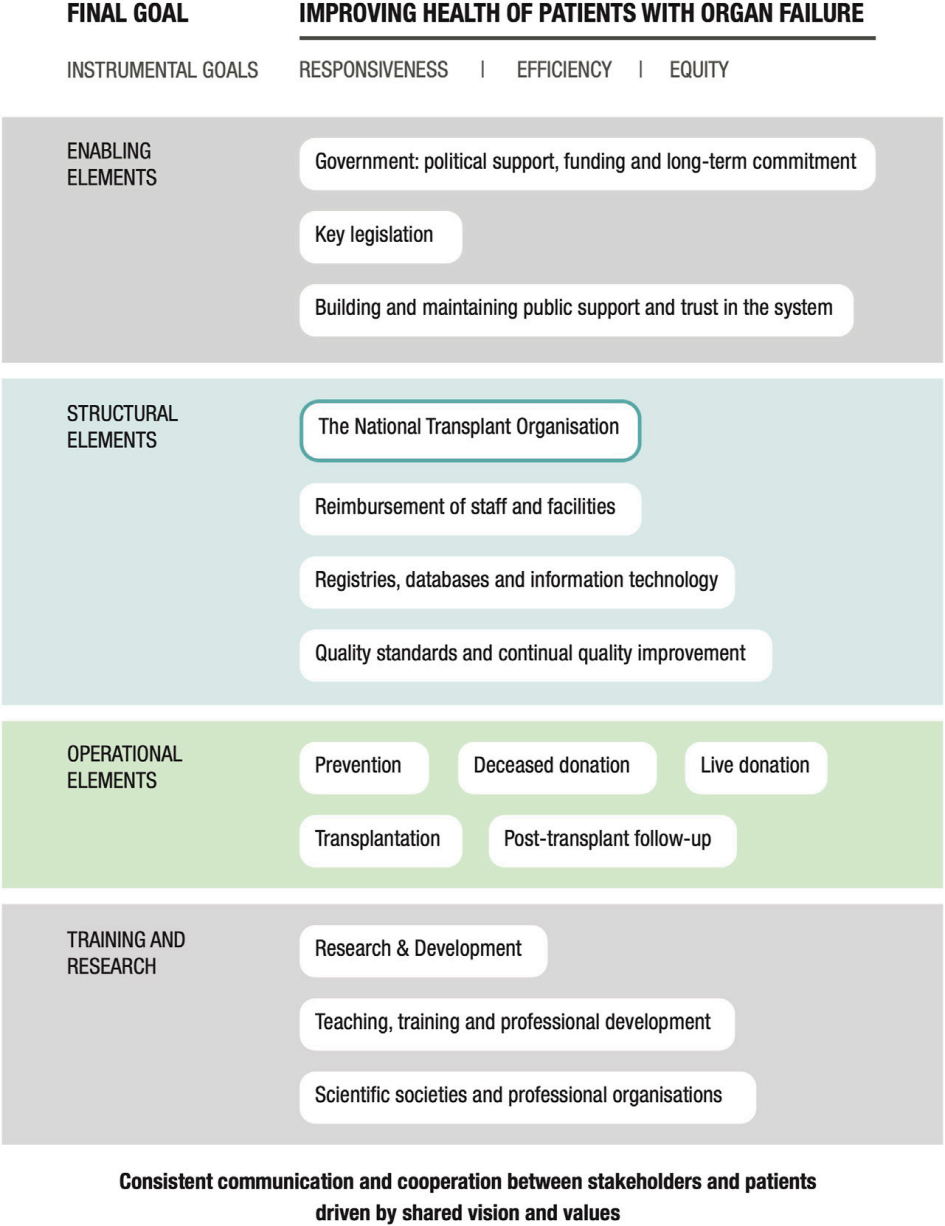
A conceptual framework for evaluating national solid organ donation and transplantation programs.

The findings were organized and coded based on the conceptual framework generated by Johnston-Webber et al. (6). The essential building blocks of an organ donation and transplantation program are depicted in Figure 1. The tables in the Results section are color-coded to match the conceptual framework. Similar to the data collection, this information was generated in multiple steps following an iterative process in which the essential building blocks of this conceptual framework were used to guide the organization of the findings that were pertinent to the Croatian system. The results were collated and verified based on findings from earlier sources of evidence. We also validated our findings in consultation with key informants and content experts who assisted us in eliminating any inconsistencies or misrepresentations. The results are presented based on critical research objectives and categorized according to the relevant essential building blocks.

## Results

### Context and Trends Identified in the Croatian Transplant System

**FIGURE 2:**
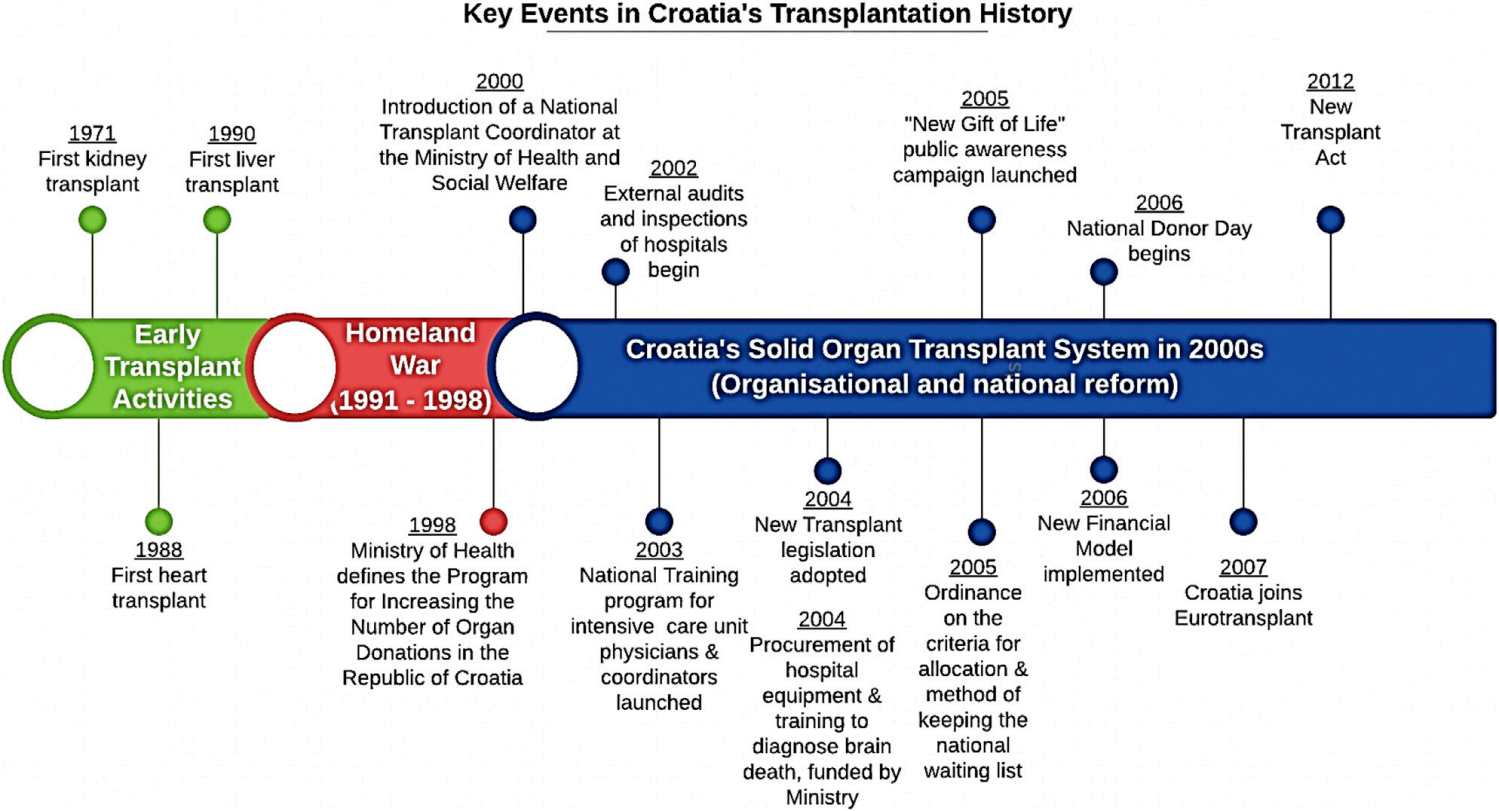
Critical events in the history of organ transplantation in Croatia.

A brief historical review will provide insight into the gains made by the Croatian transplant system (7). Croatia, formerly part of Yugoslavia, had an early interest in transplantation dating back to the 1970s. However, years of political and economic turmoil culminating in the Homeland War (1991–1998) precluded the organization of an effective organ donation and transplantation program. After the Homeland War, Croatia was left without adequate management capacity and had no national transplant or donation organization nor any vestiges of an evidence-based organ donation system. The transplant program at that time relied on outdated legislation. At that time, organ donation occurred only sporadically rather than as a routine part of clinical hospital practice. This ultimately resulted in a severe shortage of organs that could be used for transplantation (8). At the turn of the century, Croatia lagged far behind many other countries with respect to the number of organ donations and outcomes (Figure 2). To address these unmet needs and overall patient dissatisfaction, in 1998 the Ministry of Health issued an instruction designed to increase the frequency of organ donation, with priority given to organ donations from the deceased (8). One year later (1999), the Croatian Parliament passed a resolution that encouraged organ transplantation. As an adjunct to the National Transplant Organization (NTO), the ministry appointed an NTC who was charged with providing leadership and guidance in implementing the necessary reforms. This action represented a critical turning point for the development of a sustainable organ donation and transplantation system in Croatia, as it facilitated the successful implementation of new policies under the leadership of the NTC *via* a series of organizational measures that strengthened the organ donation and transplantation system.

**FIGURE 3:**
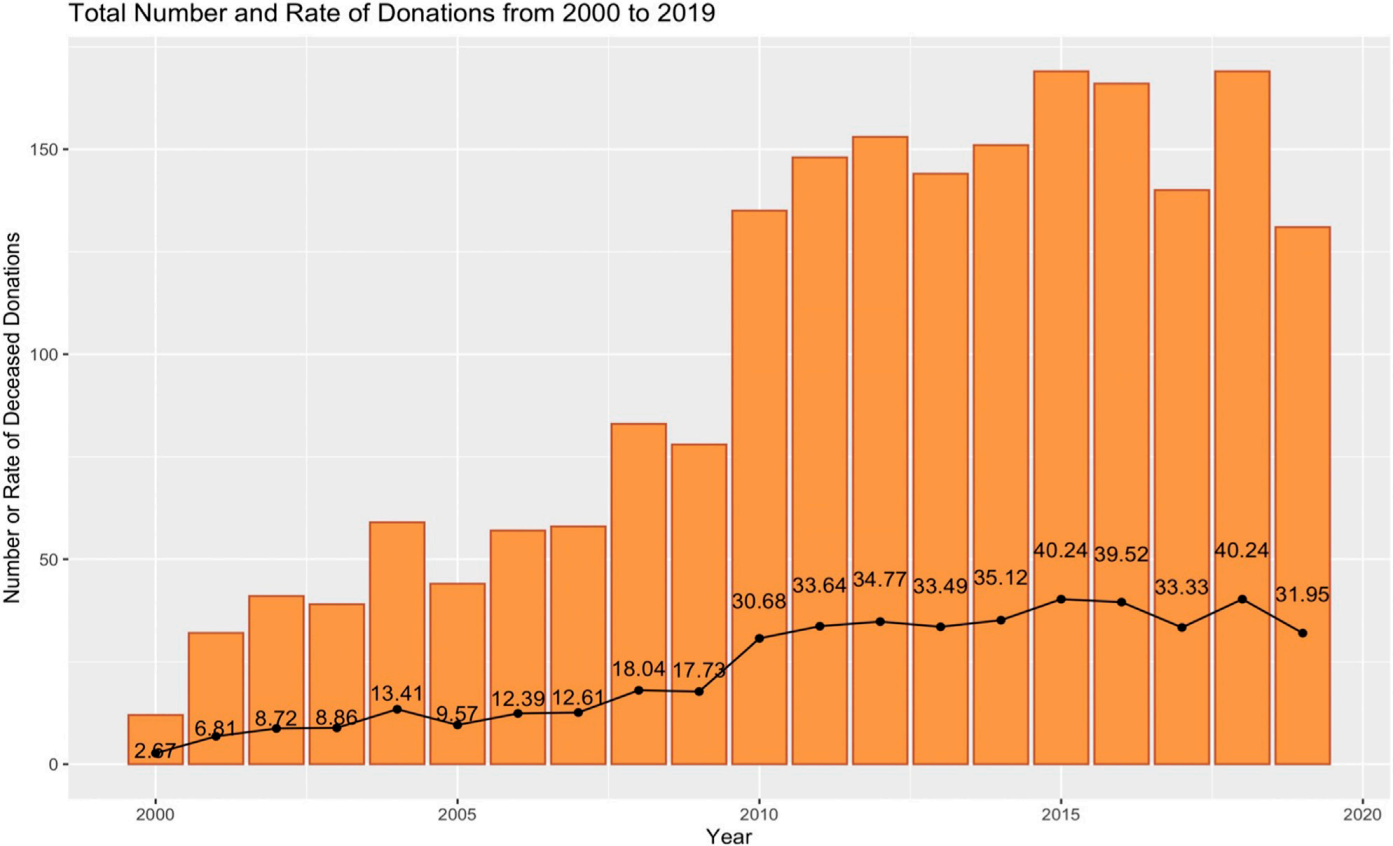
Deceased donation in Croatia from 2000 to 2019 (11). The colored bars indicate the absolute number of deceased donations; the filled circles indicate the donation rate (number per million population [pmp]).

As a result, Croatia has experienced a continuous improvement in its transplant system (Figure 3) (8). While Croatia is currently an undisputed global leader in DBD transplants, very few donations after circulatory death or living donations have been recorded since 2000 (9). Nonetheless, compared to European and global aggregated data for 2019, Croatia remains within the top 15 countries for transplant activity in terms of the number of organs and the number of patients transplanted pmp (10).

### Key Elements and Policies Leading to Transplant Reform

The following sections highlight important components that have led to the success of the Croatian organ donation and transplantation program. Table 2 shows key elements, policies, and drivers which have led to the reform of the national organ donation and transplantation program in Croatia arranged according to the conceptual framework and essential building blocks of an organ donation and transplantation program as described by Johnston-Webber et al. (11). Table 2 also highlights some features that need improvement. The information to follow is a narrative summary of several elements believed to be of particular significance.

**TABLE 2:** Key elements and policies that have led to reform of the national organ donation and transplantation program in Croatia, as reflected in domains of the conceptual framework described by Johnston-Webber et al. (11).

Framework domain	Key features	Details
Enabling Elements: Government: Political Support, Funding, Long-Term Commitment	Full political support, funding, and long-term commitment	• Government-led initiatives began in 1998 with a program designed to increase the frequency of organ donations
		• The government provided sufficient jurisdiction to the National Transplant Program enabling it to implement change
		• The government also committed financial resources to this issue (e.g., central reimbursement models for donation and transplant activities)
		See Figure 2 for additional government-supported reforms
Enabling Elements: Government: Key Legislation	Legislation implemented early on in the process of transplant reform served to reset societal expectations. This included strong DBD legislation but gaps in DCD legislation	• Early “soft” opt-out legislation (since 1988)
		• Renewal of support in 2004 *via* the Croatian Transplant Act
		• Adoption of EU quality and safety standards in 2012
		• Legislation currently does not support donation after circulatory death
Enabling Elements: Building and Maintaining Public Support and Trust in the System	Coordinated messages and inter-agency collaboration	• Continuous national campaigns, donor card promotions, and educational activities
		• Collaborations among representatives of national bodies (transplant, nephrology, and others)
Structural Elements: The National Transplant Organization (NTO)	Centralization and coordination of all aspects of donation and transplantation. Championing donation by clearly designated and expert clinical leadership	• Unique governing structure; the National Transplant Program is part of the Department of Transplantation and Biomedicine under the auspices of the Ministry of Health
		• Empowerment of an NTC and team (1999)
		• Efforts to join an international organ exchange scheme were among the early objectives of the comprehensive, long-term plan
		• Success was achieved *via* an initial focus on diagnosing brain death before moving toward an organ donation agenda
		• The National Transplant Office (NTO) is available to provide support at all times and uses creative ways to ensure adequate staffing (i.e., medical students)
Structural Elements: Infrastructure	Improvements made to immunology and histocompatibility facilities	• Two fully accredited tissue-typing laboratories were established
Structural Elements: Reimbursement of Staff and Facilities	Reimbursement by the state budget fosters local hospital participation	• Reimbursement from a special state budget rather than hospital funds
		• Adopted Transplant-related Diagnosis Related Groups
Structural Elements: Quality Standards and Continual Quality Improvement	The strong emphasis on quality improvement is provided in a helpful rather than punitive manner	• External audits and health inspections were established at major hospitals to assess the reasons underlying donor loss
		• Frontline feedback converted inspections into educational/supervisory opportunities overseen by transplant experts. This has boosted morale and the motivation to revitalize the organ donation and transplantation program
		• An official quality assurance program was established. This motivated efforts to meet the international criteria required to join Eurotransplant (2009)
Structural Elements: Registries, Databases, and Information Technology	Strengthened by efforts to join an international transplant organization	• Participation in Eurotransplant necessitated an upgrade of information technology and resulted in increased investment in the structure and transparency of transplant waiting lists and organ registries
Operational Elements: Donation	Clearly defined the role(s) of key donation personnel mandated in all public hospitals	• Critical point persons or teams overseeing donations were established (a total of 32, one in each of the public hospitals)
Operational Elements: Deceased Donation	Leader in DBD	See Figure 3
	DCD needs Improvement	• Currently, there are no DCD donors. This is a potential area for growth
Operational Elements: Live Donation	Needs improvement	• From 2000 to 2019, there were between 3 and 20 LD kidney transplants and 0–4 LD liver transplants per year (10)
Operational Elements: Transplantation	Above average for population size but relies on international collaborations for specific populations. Clearly defined roles have been established for transplantation	• 2018: Sixth globally for all transplant procedures (84.05 pmp) (12) and third globally for heart transplants (8.81 pmp) (12)
		• From 2000 to 2019, there were no lung transplants or small bowel transplants (10)
		• Pancreas transplants were performed at a rate of 1–14 per year
		• Transplant program directors/teams have been established (a total of five, all assigned to university hospitals where the procedures are performed.)
Training and Research: Teaching, Training, and Professional Development	There is a strong emphasis on training and experience	• Highly-skilled intensive care physicians provide critical support for donation programs
		• Strong emphasis on internationally- recognized training
Training and Research: Scientific Societies and Professional Organizations	International collaborations	• Involvement in multiple international transplant organizations to maintain standards and high-quality training
	Leadership of others	• Bilateral agreements provide more access for specific groups in need of transplants
		• The Croatian Model has become a leader in the South-Eastern Europe Health Network (SEEHN)

### Enabling Elements

#### Government: Political Support, Funding, Long-Term Commitment and Key Legislation

The first legislation focused on DBD donation in Croatia was enacted in 1982. This legislation was instrumental in setting normative expectations for DBD. The concept of a “soft opt-out” approach with consent for organ donation as the default option has been in place since 1988 (7, 13). New legislation (the Transplant Act) that was adopted in 2004 preserved the soft opt-out approach while adhering to the principles of the Declaration of Istanbul and Convention on Human Rights and Biomedicine (8, 14). In 2012, this legislation was harmonized with the requirements of the EU as specified in Directive 2010/53/EU which defined the quality and safety of human organs intended for transplantation, and Directive 2012/25/EU which focused on the exchange of human organs intended for transplantation between members states (14). There was no specific consideration of DCD in this set of legislative reforms. Thus, Croatia continues to rely on DBD donors alone pending any future reform efforts (9).

The aforementioned legislation was instrumental in the development of a strong and unique form of central transplant governance that was suitable for the geography and population size of Croatia. Until 2019, the National Transplant Program was under the jurisdiction of the Directorate of Transplantation and Biomedicine within the Ministry of Health of the Republic of Croatia (MZRH) (15). In 2019, the Ministry underwent an internal reorganization. As a result, decision-making and operational management of the transplant system became significantly less efficient and thus more difficult. Disruption of the governing structure, most notably during the pandemic crisis, had a negative impact on donation and transplantation rates. The observed 35% reduction in the 2019 donation rate was unlikely to be fully attributable to the pandemic as it may also be partially explained by the transition to a less effective administrative management model. Despite these concerns, Croatia remains an example of a unique, successful, and sustainable transplant system with highly effective centralized national management and leadership and strong partnerships with critically-empowered professionals and teams. This integrated model of a centralized and nationally-guided transplant system may have been established deliberately as a logical choice for Croatia, given its small geographical size, relatively homogeneous population, and limited human and financial resources.

The NTC in alliance with key donation personnel and transplant teams has played a significant role in building a sustainable transplant system.

The NTC started work in 2000 using all available legal instruments and authority of the Ministry to achieve the programme goals set forth and to introduce necessary, often "painful" changes within the health system (7;p. 56).

#### Building and Maintaining Public Support and Trust in the System

Soft opt-out legislation combined with effective public awareness and media campaigns has fostered a favorable attitude toward donation as embedded in the principle of civil solidarity and responsibility. The Ministry of Health, the Croatian Donor Network, the Croatian Transplant Association, and the Croatian Society of Nephrology, Dialysis, and Transplantation all work together to promote ongoing national campaigns, donor card promotions, and continuous educational activities (Table 2) (8). The first coordinated national public awareness campaign was launched in 2005 (7, 16). The following year, the Parliament of Croatia instituted a National Donor Day. Similarly, European Donor Day has been celebrated in Croatia since 2010 with press conferences, expert panels, and events designed to increase general public awareness of organ donation (15, 16). Organ donation is perceived as an altruistic and generous act in Croatia and is generally supported by the country’s religious communities (8).

### Structural Elements

#### The National Transplant Organization

##### Staffing and Structure

Early reform measures were aimed at promoting and optimizing deceased donation in all critical care facilities. Initially, the transplant program in Croatia relied primarily on the work of a few exceptional individuals. In 2000, the Ministry of Health established the NTC which was assigned the crucial role of driving the development of a sustainable national organ donation and transplantation program in accordance with the highest professional and international standards of practice.

The NTC has been instrumental in strengthening hospital capacity for organ donation (particularly for deceased donation) by providing additional medical education *via* a training program, systematic monitoring of performance, and round-the-clock professional and logistical support (8). The NTC provided the support and leadership necessary for the harmonization of legislation with international standards (i.e., directives from the EU, the World Health Organization, and the Council of Europe). The NTC also established a national system for monitoring hospital performance, modifying the funding model, and negotiating and preparing for international cooperation and ultimately membership in Eurotransplant (7, 8).

In addition to providing strong national governance, Croatia has also adjusted the profile and roles of key donation experts who were assigned the role of transplant coordinators to support these initiatives (16). Hospital Transplant Coordinators (HTCs) selected from the most experienced intensive care professionals undergo extensive national or international training to support their important roles in the Deceased Organ Donation Pathway. The commitment of the HTCs to deceased donation and their involvement in this pathway have provided critical support for the sustainable increases in donation rates reported by many Croatian hospitals (7). The HTCs have had a particularly important role in advocating for and promoting hospital practices that consider organ donation as an integral component of end-of-life care (7, 8).

Much of Croatia’s success may be attributed to the shared vision and efforts of the professionals involved in this pathway as well as the integrated approach that encompasses both national governance and hospital-based initiatives designed to address all essential components of organ donation and transplantation systems. In addition to the crucial role of the NTC and transplant coordinators, it is critical to recognize the contributions of intensive care teams who also provided strong support for organ donation. The efforts of these individuals were supported by transplantation and tissue typing teams. Together, these groups formed a solid foundation and represented key pillars of the modern Croatian transplant system.

##### International Collaborations

One of the NTC’s strategic goals for reform was to develop international collaborations to address the needs of the most vulnerable groups of patients. An international effort focused on high-urgency liver transplants was initiated in 2004 and 2005 *via* a collaboration with the Italian Transplant Network (Centro Nacionale Trapianti).

In 2006, once all the key pillars of a successful transplant program had been achieved (e.g., a donor rate above 10 pmp, an around-the-clock central service, and tissue typing laboratory accreditation), the NTC approached the Eurotransplant Board of Management and commenced the negotiation process for membership in Eurotransplant. In 2007, once additional improvements and harmonization with international standards had been achieved, Croatia joined Eurotransplant. This was understood to be a critical step toward achieving quality and excellence, particularly in the fields of immunogenetics (7, 13), organ allocation, and utility of donated organs (7). Membership in Eurotransplant was also viewed as a means of increasing the public trust in the integrity of the Croatian national transplant program. This action had the additional indirect effect of motivating the key donation personnel, most notably critical care teams:

Namely, the loss, i.e. rejection of "marginal" organs as unacceptable or even rejection of good quality organs, used to be a "privilege" of our transplant centres, which often resulted in revolt and dissatisfaction of hospital transplant coordinators. In what was no doubt an attempt to find "ideal" organs for their recipients, transplant teams often rejected marginal organs, as unacceptable, even in times of organ shortage. Upon joining Eurotransplant, this practice as a result from a "monopolistic" position and lack of competition (in cases where only one transplant centre existed) and a lack of any kind of supervision over ethical justification of such decisions, almost completely disappeared (7; p. 64).

As more organ donations materialized, it became easier to manage the waiting list. Patients on waiting lists were also provided with better screening, organ matching, and pre-operative evaluation, all of which resulted in better transplant outcomes (7).

### Reimbursement of Staff and Facilities

As part of the overall effort to counteract the lack of financial resources, the limited number of appropriately-trained health professionals, and inadequate salaries associated with the donation and transplantation program in individual hospitals, new reimbursement schemes were adopted. Beginning in 2006, compensation for organ donation was provided by a special state budget; this ensured that the process involved no financial burden to local hospitals. The cost of the transplant evaluation, assessment, procedures, and operations are paid through Diagnosis Related Groups formulated by HZZO (7). On average, the 70,000–350,000 HRK (Croatian kuna) reimbursement for transplantation (depending on the complexity of a given procedure) is paid directly to the transplant center. Each donor hospital receives 40,000–55,000 HRK to cover the costs of donor recruitment, preparation, and organ retrieval (7). This reimbursement strategy has successfully promoted organ donations at smaller hospitals without transplant programs and has promoted the identification and support of potential donors.

### Quality Standards and Continual Quality Improvement

Accountability mechanisms were established early on during this process and include audits and expert supervision. The design of these interventions was based mainly on current methods of health inspection and the Spanish donor quality assurance program. The first external audits and inspections were conducted in 2003 in a selected group of larger hospitals. The goal of these activities was to gain some understanding of the actual rate of brain death and the specific reasons underlying donor loss. This initial approach was upgraded and improved several times since then. A more systematic quality assurance program for DBD donors was introduced in 2009 that included annual desk-based audits. Depending on inspection capacities, on-site audits are conducted every 2 years to assess the performance of deceased organ donation processes. The inspection commission includes a health inspector, the NTC, and selected prominent HTCs. These inspections focus increasingly on educational and motivational strategies, as this method is preferred by the hospital staff. This “soft” approach to quality assurance helps to motivate the staff regarding the implementation of positive change and toward eliminating previously-identified gaps in the deceased donation pathway (7). Transplantation outcomes are assessed separately in each transplant program *via* regular audits of transplant centers at two-year intervals.

### Remaining Challenges

The Croatian organ donation and transplantation program has achieved undeniable success. Croatia has contributed to a regional initiative and provides technical assistance designed to promote the development of sustainable transplant systems in neighboring South-Eastern European countries, including Serbia, North Macedonia, Moldova, Bosnia and Herzegovina, Romania, Bulgaria, Montenegro, and Albania). The results of this collaboration, which was spearheaded by the Croatian NTC’s Regional Health Development Centre on Organ Donation and Transplant Medicine, show that these countries have all experienced improvements in their organ transplant activities (2011–2015) (17). However, it is important to recognize that one-third to one-half of the donations in several of these countries are from DCDs and LDs.

The gaps in the Croatian program are delineated by the framework approach. At this time, there are no DCD donations in Croatia and very few from LDs (Table 2). To enlarge the donor pool, Croatia is considering the development of a controlled DCD (cDCD) program (17). While the Croatian organ donation and transplantation program currently has the infrastructure that would be needed to implement this type of program, it will require additional staff training and a proper legal framework. These components are currently in development (17).

There is always work to be done to maintain public trust in the system and willingness to donate. According to the latest Eurobarometer survey on organ donation, Croatia is above the European average for knowledge of national regulations regarding organ donation and transplantation, and of discussing human organ donation (18). Croatia (53%) is almost at the European average (55%) for willingness to donate own organs. One explanation for positive donation sentiment is that Croatia is a very homogenous country with 90.4% of the population being Croats, 95.6% of the population identifying Croatian as their mother tongue and ∼ 91.4% being Christians (the vast majority Catholics) (19). This offers an advantage when it comes to building a pro-donation attitude in society and when it comes to approaching families for donation. However, compared to 53% of Europeans, only 45% of Croatians would be willing to do donate a family member’s organs. Reasons not to donate a family’s organs include above average fears of manipulation of the human body (18). A larger proportion of Croatians did not have a reason for unwillingness to donate, suggesting an opportunity for initiatives building trust (described above) with the general population (18).

## Discussion

This review summarizes the key aspects of the Croatian transplantation system and has highlighted factors that have contributed to its remarkable success. The results of our evaluation are presented with reference to a comprehensive framework that includes system-level elements that are essential for a successful organ donation and transplantation program (6). The case study specifically highlights several components that are of particular importance to the Croatian program. These include landmark legislation, central reimbursement for organ donation and transplantation activities, ongoing public awareness campaigns, and recruitment of skilled intensivists to serve as critical donation personnel. Croatia has achieved success with a unique model that includes strong central governance and the appointment of an empowered national clinical leader whose role is overseen directly by the Ministry of Health. In 2007, Croatia realized its ambition of joining Eurotransplant. To meet these requirements, Croatia needed to strengthen several features of its donation and transplantation program, including factors involved in its infrastructure, registries, and processes.

There remain large gaps in transplantation research at the systems level, particularly with respect to the complex relationships between politics, legislation, and public opinions regarding organ donation and transplant activities. A more complete understanding of these system-level relationships may help us to understand why one transplant program has succeeded while others have failed. The use of a comprehensive, systems-level framework such as the one employed here (11) may help to identify specific areas of strong *versus* poor performance. Our findings present considerations that may be useful to countries that are similar to Croatia in terms of population size and/or financial resources. Croatia is an excellent example of how a small and poorly-resourced country can achieve success in organ donation and transplantation. Our evaluation offers a wealth of information and potential strategies that might be utilized to transform a low-performing into a high-performing program.

This paper builds on previous publications that describe the Croatian organ donation and transplantation model and actions taken that have boosted donation rates (7, 8, 20). However, this analysis is unique because it is the first to explore aspects of governance and policy that might be adapted for use by other nations seeking to develop transplant systems. A major strength of this paper is that the findings were based on multiple sources of information and thus captured a holistic, detailed picture of the system in a real-life context.

We recognize that there are limitations to the chosen methodology. Of note, we understand that the evidence provided may be difficult to generalize and that our findings may not apply to all other contexts and situations. We believe that these concerns are somewhat mitigated by our approach to data collection and analysis, including the use of a conceptual framework and validation of our findings by subject matter experts. We do recognize that the potential bias introduced by these experts may also be a limitation of this work and that their views may not be relevant to all transplant situations. However, given the significant gap in the literature on the nature of successful transplant systems, we hope that these findings, together with the contributions of other reviews in this series, contribute to future research in this area. Additional comparative research focused on the transplant systems in other countries helps to strengthen our understanding of the factors identified as crucial to successful organ donation and transplantation programs. This information is provided by several other papers in this series (21–25).

We were unable to locate full information for several of the domains identified in the conceptual framework. For example, efforts designed to prevent the need for an organ transplant are largely outside the purview of the Croatian organ donation and transplantation program. Likewise, we found very little information that addressed post-transplant follow-up in Croatia or the barriers preventing DCD or LD. However, the use of the framework was helpful as it permitted us to identify these areas as subjects for future research and quality improvement initiatives.

In summary, the Croatian experience demonstrates that countries with relatively modest resources can successfully build world-class organ donation and transplantation systems. Effective processes identified in this study include strong central governance as well as effective leadership at both the political (government and legislation) and clinical (champions and key donation persons) levels. Additionally, the act of joining an international organ exchange scheme helped to guide the transformation process and drive improvements in program quality.

## Data Availability

The original contributions presented in the study are included in the article/supplementary material, further inquiries can be directed to the corresponding author.
